# *Tspo* Depletion Exacerbates Steatosis Through Fatty Acid Uptake

**DOI:** 10.1111/jcmm.70500

**Published:** 2025-04-07

**Authors:** Yuchang Li, Liting Chen, Chantal Sottas, Nrupa Dinesh Patel, Mahima Chandrakant Raul, Vassilios Papadopoulos

**Affiliations:** ^1^ Department of Pharmacology and Pharmaceutical Sciences, Alfred E. Mann School of Pharmacy and Pharmaceutical Sciences University of Southern California Los Angeles California USA

**Keywords:** acetyl‐coenzyme a, de novo lipogenesis, fatty acid oxidation, MASLD, RAPTOR

## Abstract

Previous studies demonstrated that *Tspo* loss causes simple steatosis (SS) in hepatocytes in vitro. However, its effect on SS in vivo remains unclear. In this study, we hypothesise that *Tspo* loss promotes early‐stage MASLD. WT and *Tspo* KO rats were fed a Gubra Amylin NASH (GAN) diet for 8 weeks to induce SS. *Tspo* KO rats fed the GAN diet (KO GAN) exhibited increased insulin resistance, higher plasma cholesterol, and elevated hepatic triacylglycerol (TAG) levels, along with higher de novo lipogenesis (DNL) and free fatty acid (FFA) uptake, evidenced by increased fatty acid synthase (FASN) and CD36 expression. The Acyl‐coenzyme A binding protein/diazepam‐binding inhibitor‐TSPO complex facilitated FA transport to the mitochondria, where carnitine palmitoyltransferase 1A (CPT1A) directed them for β‐oxidation. TSPO interacted with CPT1A in the outer mitochondrial membrane, while its depletion increased CPT1A expression, boosting FA oxidation. Primary *Tspo* KO rat hepatocytes and stably overexpressed CD36 (CD36_OE) in Huh7 cells displayed impaired mitochondrial function and compromised mitochondrial membrane potential. KO GAN livers had significantly elevated AcCoA, which acetylated RAPTOR, activating mTORC1 to suppress autophagy. Overall, *Tspo* deficiency exacerbates the advancement of SS by enhancing CD36‐mediated FFA uptake, elevating AcCoA levels, compromising mitochondrial function and impairing autophagy during the early stages of MASLD.

**Abbreviations**
ACBP/DBI: acyl‐coenzyme A binding protein/diazepam‐binding inhibitor
AcCoA: acetyl‐CoA
ACOX1: acyl‐CoA oxidase 1
CD36: cluster of differentiation 36
Co‐IP: co‐immunoprecipitation
CPT1A: carnitine palmitoyltransferase IA
CYP7A1: cytochrome P450 family 7 subfamily A member 1
CYP27A1: cytochrome P450 family 27 subfamily A member 1
DNL: de novo lipogenesis
FA: fatty acid
FFAs: free fatty acids
FASN: fatty acid synthase
GAN diet: Gubra‐Amylin NASH diet
GTT: glucose tolerance test
ITT: insulin tolerance test
MASLD: metabolic dysfunction‐associated steatotic liver disease
PLA: proximity ligation assay
PPARα: peroxisome proliferator‐activated receptor alpha
RPS6: ribosomal protein S6
SREBP: sterol regulatory element‐binding protein
TAG: triacylglycerol
TNFα: tumour necrosis factor alpha
ULK1: UNC‐51‐like autophagy‐activating kinase 1
VDAC1: voltage‐dependent anion channel 1
VLDL: very low‐density lipoprotein

## 1 Introduction

Metabolic dysfunction‐associated steatotic liver disease (MASLD) is a significant liver condition involving multiple bodily systems and is recognised as the hepatic manifestation of metabolic syndrome (MetS) and insulin resistance (IR) [1]. MASLD encompasses a spectrum of severity, ranging from benign hepatocellular simple steatosis (SS), characterised by lipid accumulation in hepatocytes, to metabolic dysfunction‐associated steatohepatitis (MASH), marked by liver inflammation, fibrosis, and cell death, and ultimately progressing to irreversible stages such as cirrhosis and hepatocellular carcinoma (HCC). While the Food and Drug Administration (FDA) has approved the first drug, resmetirom (Rezdiffra), for treating advanced stages of MASH [2], understanding the underlying mechanisms initiating early stages of MASLD remains elusive.

The translocator protein (TSPO, 18 kDa), located in the outer mitochondrial membrane (OMM), binds and transports cholesterol from the OMM to the inner mitochondrial membrane (IMM) for hormone synthesis in steroidogenic cells or bile acid (BA) synthesis in the liver [3, 4, 5]. We and other groups have demonstrated that TSPO is a potential marker for MASLD progression [5, 6, 7]. In human hepatocytes in vitro, *TSPO* deficiency accelerated SS following lipid loading by instigating a cascade of signalling events, including lipid droplet enlargement and endoplasmic reticulum (ER) stress [5]. However, in methionine choline‐deficient diet (MCDD)‐induced MASH, loss of *Tspo* ameliorated the MASH via reduced BA biosynthesis through downregulation of cytochrome p450 family 7 subfamily A member 1 (CYP7A1) and cytochrome p450 family 27 subfamily A member 1 (CYP27A1) and upregulation of farnesoid x receptor (FXR) [5], a well‐recognised therapeutic target of MASH [8]. Recently, we demonstrated that the TSPO ligand Atriol mitigates MASH by downregulating CXC motif chemokine ligand 1 (CXCL1) [9]. These investigations suggested that TSPO plays a multifaceted role in the development of MASLD.

In the early stages of MASLD, extensive research has elucidated the various sources of fat contributing to hepatic steatosis, encompassing de novo lipogenesis (DNL), the transport of plasma free fatty acids (FFA) derived from adipose tissue, fatty acid oxidation (FAO) in hepatocytes, and the secretion of triacylglycerols (TAGs) via very low‐density lipoproteins (VLDLs) [10, 11]. The liver's condition is dictated by dynamic fluctuations in the rates of DNL, FAO, FFA import, and TAG export. When the rates of DNL and FFA imports surpass FAO and TAG export, hepatic fat accumulation occurs. Oversight of these metabolic shifts primarily falls upon sterol regulatory element‐binding proteins (SREBPs), the master regulators of lipogenesis and lipid metabolism. SREBPs directly activate over 30 genes dedicated to the synthesis and uptake of cholesterol, fatty acids (FA), TAGs and phospholipids [12]. Notably, while SREBP‐1a and SREBP‐1c predominantly influence genes involved in FA synthesis [13, 14], SREBP‐2 activates genes responsible for cholesterol synthesis [12].

This study explores the involvement of TSPO in the development of SS induced by a Gubra‐Amylin NASH (GAN) diet in rats. Our investigation reveals that *Tspo* deficiency exacerbates SS, primarily attributable to increased DNL, cellular uptake of FFA and subsequent inhibition of autophagy through mammalian target of rapamycin complex 1 (mTORC1) activation by acetylated regulatory‐associated protein of mTOR (RAPTOR). These findings underscore the pivotal role of TSPO in preserving lipid homeostasis. The absence of *Tspo* renders rats vulnerable to hepatic lipotoxicity stemming from perturbed lipid balance and diminished autophagic activity.

## 2 Materials and Methods

### 2.1 Experimental Animals

All animal protocols were approved by the IACUC of the University of Southern California. *Tspo* KO rats were generated using CompoZr knockout Zinc Finger Nuclease Technology (CKOZFN64723; Sigma–Aldrich, Saint Louis, U.S.A.) to delete an 89‐bp fragment flanking the exon 3/intron 3 junction in the *Tspo* gene, and the rats were genotyped using previously described primer sets [15].

WT and *Tspo* KO male rats aged at postnatal day (PND) 50 were fed with Gubra‐Amylin NASH (GAN) (40 kcal% fat, 20 kcal% fructose and 2% cholesterol; #D09100310, Research Diets, New Brunswick, NJ, USA) or low‐fat diet (LFD; #D09100304, Research Diets, New Brunswick, NJ, USA) for 8 weeks under a 12/12 h light/dark cycle at room temperature (21°C ± 2°C). Water was available *ad libitum*. The body weight was measured every week, and food intake was recorded daily. The animals were divided into 4 groups: WT LFD, *Tspo* KO (KO) LFD, WT GAN, and *Tspo* KO (KO) GAN (*n* = 10 each group).

### 2.2 The Other Detailed Materials and Methods Are Provided as Materials and Methods in Supplemental Information

### 2.3 Statistical Analysis

Data analyses were performed with GraphPad Prism 10.2.0 software. The normal (Gaussian) distribution was assessed with the Shapiro–Wilk test (*n* = 3 or small size) or D'Agostino & Pearson test (*n* > 6 or large size) and visualised using QQ plots. The parametric analysis was applied for one‐way ANOVA followed by Tukey's multiple comparisons. *p* < 0.05 was considered statistically significant.

## 3 Results

### 3.1 Lipid Accumulation and Insulin Resistance in *Tspo* KO GAN Rats

**FIGURE 1:**
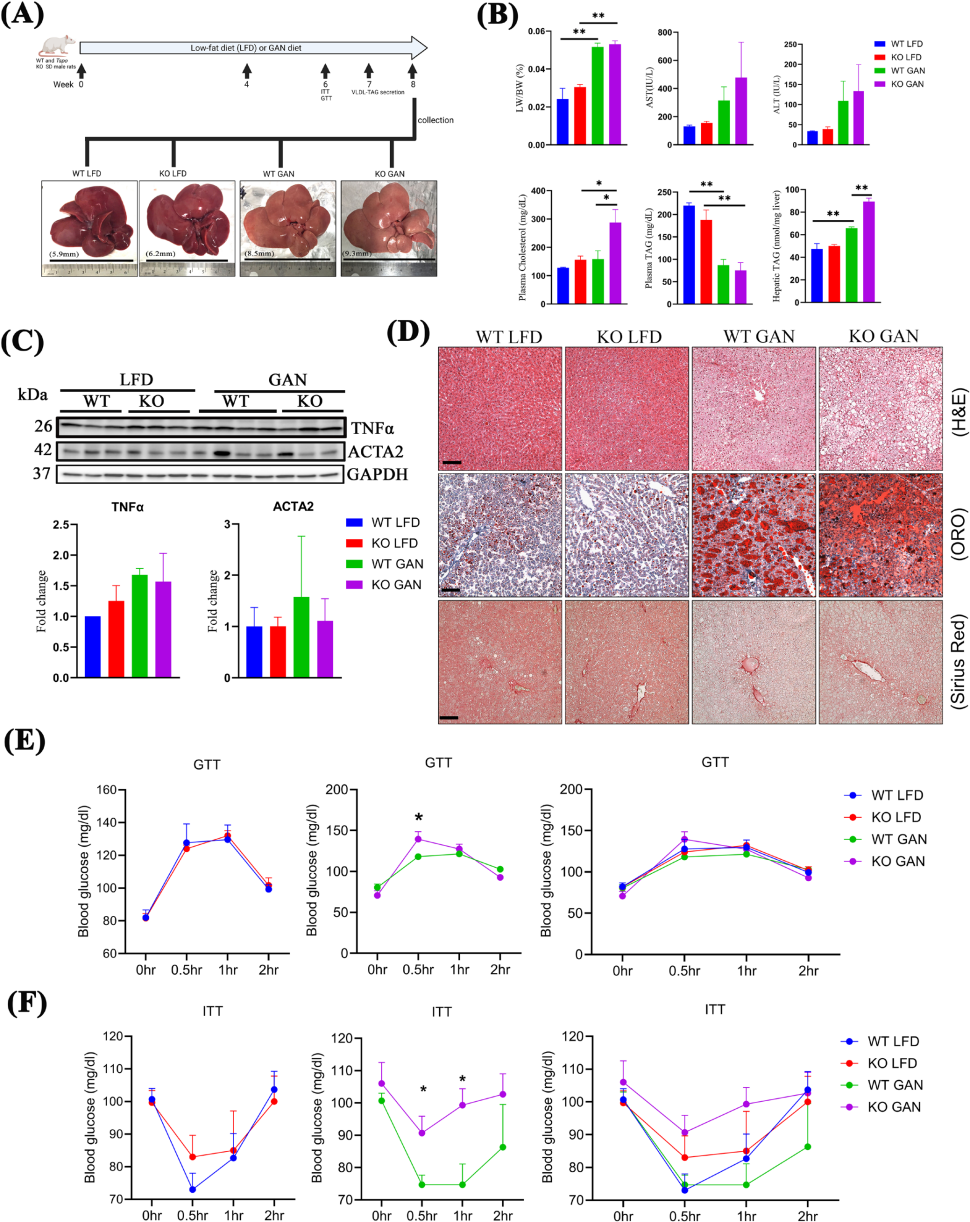
Lipids accumulation and insulin resistance in *Tspo* KO with GAN diet. (A) WT and *Tspo* KO rats were fed either a GAN diet or a low‐fat diet (LFD) over an 8‐week period. During the 7 weeks, GTT, ITT, and VLDL‐TAG were performed. The rats were divided into four groups: WT LFD, KO LFD, WT GAN, and KO GAN. The representative liver images were taken when collection. (B) Liver weight and body weight ratio (LW/BW), AST, ALT, plasma cholesterol. Plasma TAG, and hepatic TAG were measured (*n* = 10). (C) Immunoblot analysis of TNFα and ACTA2 with normalisation by GAPDH (*n* = 3). (D) Representative histological images of the livers by H&E staining, Oil Red O staining, and Sirius red staining (*n* = 3). Scale bar: 100 μm. GTT in (E) (*n* = 10) and ITT in (F) were performed (*n* = 10). ACTA2, actin alpha 2 (smooth muscle); ALT, alanine aminotransferase; AST, aspartate aminotransferase; GAN, Gubra‐Amylin NASH; GAPDH, glyceraldehyde 3‐phosphate dehydrogenase; GTT, glucose tolerance test; H&E, haematoxylin and eosin; ITT, insulin tolerance test; Oro, oil red o; TAG, triacylglycerol; TNFα, tumour necrosis factor alpha; VLDL‐TAG, very low‐density lipoprotein TAG. Data are presented as mean ± SEM. *p < 0.05, **p < 0.01 by one‐way ANOVA.

To investigate the potential involvement of TSPO in the progression of MASLD, WT and *Tspo* knockout (KO) rats were subjected to an experimental regimen involving either a GAN diet or a low‐fat diet (LFD) over an 8‐week period. The rats were categorised into four distinct groups: WT LFD, KO LFD, WT GAN, and KO GAN (Figure 1A). Throughout the feeding, there were no discernible differences in food consumption between WT and KO rats, regardless of diet composition (Figure S1). However, a marked increase in liver length and liver weight‐to‐body weight ratio (LW/BW) was observed following GAN diet administration in both WT and KO animals (Figure 1A,B), confirmation of the development of fatty liver. Notably, there was no notable disparity in LW/BW between the WT and KO GAN cohorts. Evaluation of alanine transaminase (ALT) and aspartate transaminase (AST), recognised as enzymatic markers for liver damage, revealed a tendency towards elevation in the KO GAN group relative to the WT GAN, albeit not reaching statistical significance (Figure 1B). Concurrently, plasma cholesterol and hepatic triglyceride (TAG) levels exhibited significant increases, while plasma TAG levels remained comparable between the WT and KO GAN groups (Figure 1B). Immunoblot analysis indicated upregulation in TSPO levels in WT rats following GAN diet (Figure S2), aligning with our previous study on the development of MASLD [5]. Despite these changes, inflammatory marker TNFα and liver fibrosis marker ACTA2 manifested no significant alterations across the four experimental groups, suggesting that 8 weeks of GAN diet feeding instigated SS, but not MASH (Figure 1C). Consistently, Sirius red staining revealed no evidence of fibrosis across the livers among these four experimental groups (Sirius red staining in Figure 1D). Histological assessment unveiled a broader spectrum of steatosis following GAN diet administration, with a more pronounced accumulation of fat vacuoles observed in the KO group compared to WT (H&E staining in Figure 1D). Oil Red O (ORO) staining corroborated the accrual of lipids induced by GAN diet feeding, with a more pronounced effect observed in the KO group relative to WT (ORO in Figure 1D). Furthermore, we treated primary hepatocytes isolated from WT and *Tspo* KO rats with oleic acid (OA) and measured TAG content; the results suggested that TAG was significantly increased after OA treatment in WT hepatocytes but increased more in KO cells (Figure S3).

To evaluate the impact of *Tspo* loss on metabolic effects following a GAN diet, we conducted glucose tolerance tests (GTT) and insulin tolerance tests (ITT). We observed no significant difference between WT LFD and KO LFD. However, there was reduced blood glucose tolerance during the GTT at 0.5 h in KO GAN compared to WT GAN in response to glucose administration to fasting rats (Figure 1E). This finding was further supported by the ITT results, which showed higher blood glucose levels at 0.5–1 h following insulin injection in the KO rats, suggesting insulin resistance in KO rats (Figure 1F). Collectively, these findings suggest that 8 weeks of GAN diet led to SS, with *Tspo* deficiency exacerbating this pathological process.

### 3.2 Higher DNL and Fatty Acid Uptake in *Tspo* KO Rats Under a GAN Diet

**FIGURE 2:**
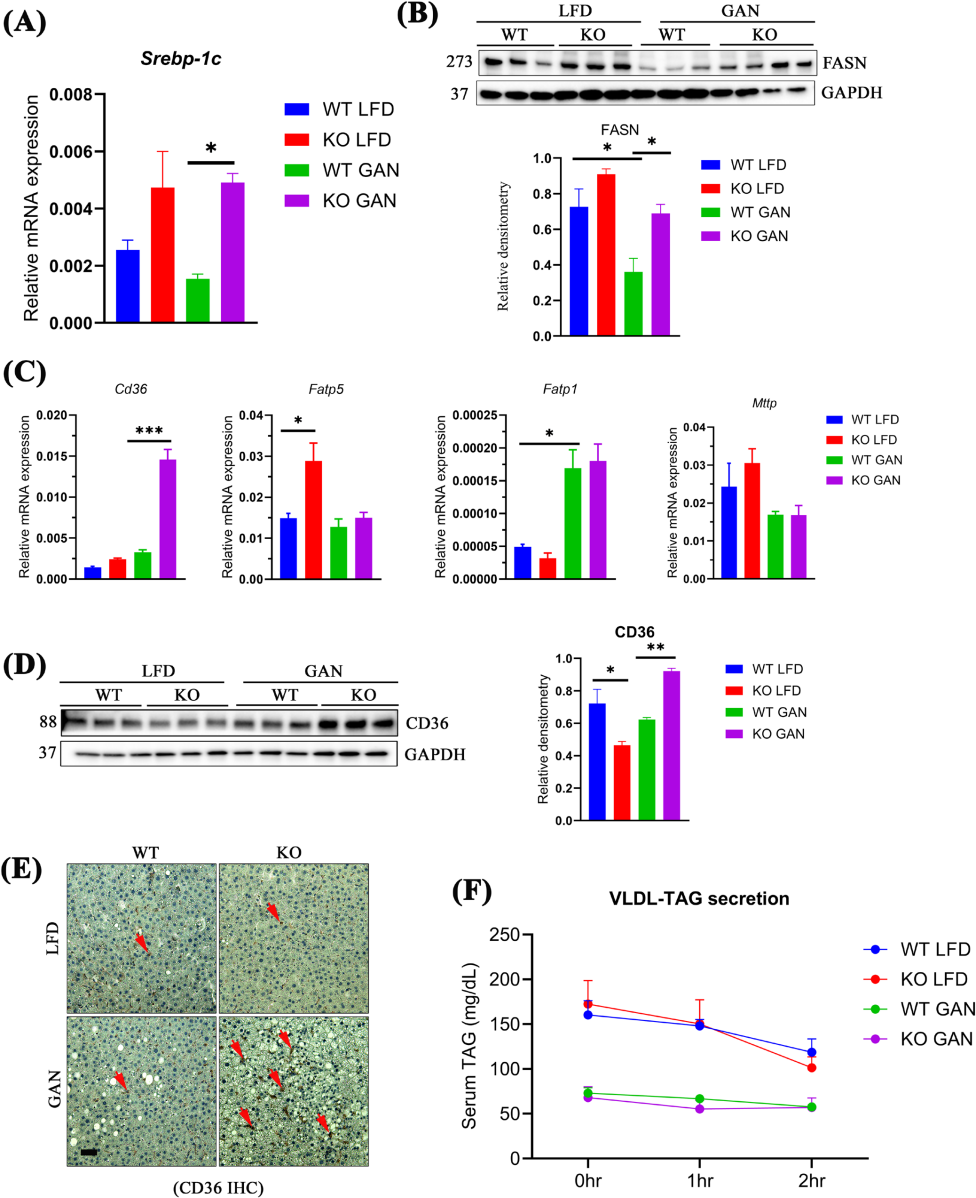
Higher DNL and fatty acid uptake in *Tspo* KO compared to WT with GAN diet (A) qPCR analysis of *Srebp‐1c* (*n* = 3). (B) Immunoblot analysis of FASN and quantification (*n* = 3). (C) qPCR of *Cd36, Fatp5, Fatp1*, and *Mttp* (*n* = 3). (D) Immunoblot analysis of CD36 (*n* = 3). (E) Immunohistochemistry staining of CD36 (*n* = 3), red arrow indicated positive signal. (F) Serum TAG measurement for VLDL‐TAG secretion (*n* = 10). DNL, de novo lipogenesis; FASN, fatty acid synthase; Cd36, cluster of differentiation 36; Fatp, fatty acid transport protein; GAN, Gubra‐Amylin NASH; Mttp, microsomal triglyceride transfer protein; Srebp‐1c, sterol regulatory element‐binding protein‐1c; VLDL‐TAG, very low‐density lipoprotein triacylglycerol. Scale bar: 20 μm. Data are presented as mean ± SEM, *p < 0.05, **p < 0.01, ***p < 0.001 by one‐way ANOVA.

Next, we investigated alterations in pivotal enzymes responsible for DNL and very low‐density lipoprotein‐triacylglycerols (VLDL‐TAGs) secretion from the liver, two main processes in the regulation of TAG homeostasis. SREBP‐1c is the master regulator of lipid synthesis for DNL, which contributes to TAG build‐up [13, 14]. After GAN feeding, *Srebp‐1c* mRNA was substantially increased in KO compared to WT with a GAN diet, implying heightened DNL in KO animals (Figure 2A). Fatty acid synthetase (FASN), a key terminal regulator in DNL responsible for the biosynthesis of long‐chain saturated FAs [16], exhibited a significant increase in KO compared to WT GAN animals (Figure 2B). These data suggest that the absence of TSPO intensifies TAG accumulation upon GAN exposure. Meanwhile, we explored whether cellular fatty acid (FA) import contributes to lipid accumulation. Notably, the mRNA levels of *Cd36*, a crucial membrane glycoprotein facilitating FA uptake, exhibited significant increases in KO GAN compared to WT GAN animals. However, no alterations were observed in *Fatp5/Slc27a5* (fatty acid transporter protein 5/solute carrier family 27 member 5), *Fatp1*, or the TAG export marker microsomal triglyceride transfer protein (*Mttp*) between WT and KO GAN (Figure 2C). Consistently, immunoblot analysis and immunofluorescence staining confirmed elevated CD36 expression in KO GAN compared to WT GAN (Figure 2D,E). Furthermore, assessing in vivo VLDL‐TAG secretion showed reduced secretion after the GAN diet compared to LFD. However, minimal differences were observed between the WT GAN and KO GAN groups (Figure 2F). Collectively, these observations indicated that the steatotic liver condition in KO rats primarily arises from increased fatty acid import and DNL, but not from TAG secretion.

### 3.3 *Tspo* Deficiency Enhances the Transportation of Fatty Acids to the Mitochondria for FAO

**FIGURE 3:**
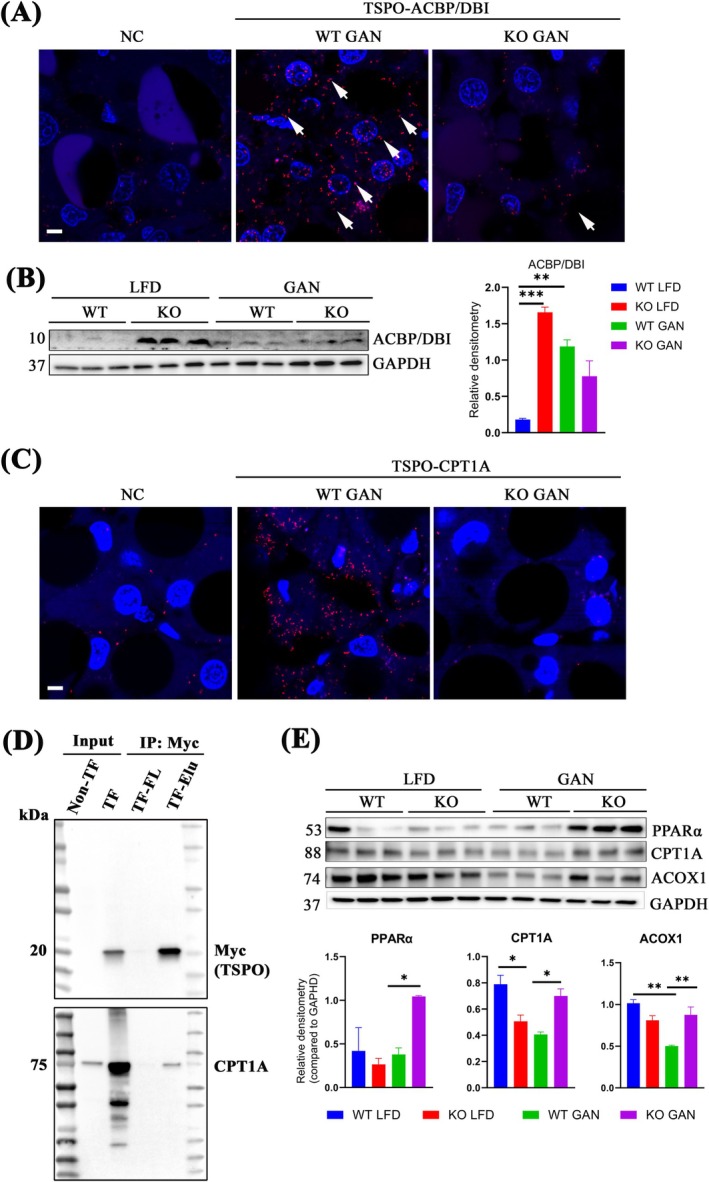
Fatty acid transportation to mitochondria for FAO though fatty acyl‐CoA‐CPT1A. (A) PLA of TSPO and ACBP/DBI in liver tissues, white arrows indicated positive signal (red). NC is negative control no adding primary antibody. (B) Immunoblot analysis of ACBP/DBI and quantification (*n* = 3). (C) PLA of TSPO‐CPT1A in liver tissue; white arrows indicate red signal. NC is negative control no adding primary antibody. (D) Human *Tspo*‐Myc plasmid was transfected into H293 cells. The cell pellets were collected for immunoprecipitation by Myc‐Trap agarose beads. Immunoblot analysis with anti‐Myc and anti‐CPT1A antibodies was performed. Non‐TF: Non‐transfection. TF: Transfection. TF‐FL: Flow through from transfected cells. TF‐Elu: Elutes from transfected cells. (E) Immunoblot analysis in the liver of PPARα, CPT1A, and ACOX1 and quantification (*n* = 3). Scale bar: 20 μm in (A) and (C). FAO: Fatty acid oxidation; acyl‐CoA: Acyl‐Coenzyme A; CPT1A: Carnitine palmitoyltransferase 1A; PLA: Proximity ligation assay; ACBP/DBI: Acyl‐CoA binding protein/diazepam binding inhibitor; IP: Immunoprecipitation; PPARα: Peroxisome proliferator‐activated receptor alpha; ACOX1: Acyl‐CoA oxidase 1. The same immunoblot membrane was used for both Figures 2D and 3B, with GAPDH as the common loading control. Data are presented as mean ± SEM, *p < 0.05, **p < 0.01, ***p < 0.001 by one‐way ANOVA.

CD36, situated in the hepatocyte plasma membrane, is associated with elevated steatosis, involving increased circulating FFA uptake and subsequent TAG storage in the liver [17, 18]. To delineate the fate of fatty acids after uptake by CD36 in hepatocytes, we investigated the change of Acyl‐CoA binding protein (ACBP), also known as diazepam‐binding inhibitor (DBI), an endogenous binding ligand of TSPO [19], which plays a crucial role in binding medium and long‐chain fatty acyl‐CoAs (M‐LCACoA) and facilitates the transport of these fatty acids from the cytoplasm to the mitochondria, thereby enhancing fatty acid oxidation (FAO) [20]. Firstly, we confirmed this interaction between TSPO and ACBP/DBI through proximity ligation assay (PLA) on liver tissue visualised in red spots (white arrows) in WT GAN, whereas no signal was detected in KO GAN (Figure 3A). Immunoblot analysis revealed a significant upregulation of ACBP/DBI in *Tspo* KO compared to WT with LFD. However, even though ACBP/DBI levels were notably increased following the GAN diet, there was no discernible difference between WT and KO with GAN (Figure 3B).

Mitochondria play a pivotal role in either supporting fatty acid synthesis or oxidation through the regulation of fatty acid entry, primarily controlled by the carnitine palmitoyltransferase (CPT) system [21]. Within this system, carnitine palmitoyltransferase 1 (CPT1) serves as a critical transmembrane enzyme located on the OMM, which converts long‐chain fatty acyl‐CoA to long‐chain acylcarnitine, following which carnitine acylcarnitine translocase transports the fatty acid across the IMM and then enters fatty acid β‐oxidation [22, 23]. Previous work has demonstrated the association of CPT1A with voltage‐dependent anion channel 1 (VDAC1), another OMM protein [24, 25]. VDAC1 is known to form complexes with TSPO [26]. However, whether TSPO interacts with CPTA1 needs to be explored. To do so, we conducted PLA for TSPO and CPT1A on WT GAN and *Tspo* KO GAN liver slides. The results revealed abundant red spot signals in WT GAN livers but no signal in *Tspo* KO livers (Figure 3C), suggesting a potential interaction between TSPO and CPT1A. Further, we transiently transfected human TSPO‐DDK‐Myc plasmid into HEK293, a human embryonic kidney cell line, followed by immunoprecipitation using Myc‐Trap agarose beads. Immunoblot analysis with anti‐Myc and anti‐CPT1A antibodies showed Myc (indicative of TSPO) and CPT1A bands only in the eluted fraction in TSPO‐DDK‐Myc transfected cells (Figure 3D). These data suggest an interaction between TSPO and CPT1A in the OMM.

Subsequently, we investigated the key enzymes involved in FAO. Immunoblot analysis revealed a significant increase in the expression levels of peroxisome proliferator‐activated receptor α (PPARα), CPT1A, and peroxisomal acyl‐CoA oxidase 1 (ACOX1) in KO GAN compared to WT GAN (Figure 3E). This implies that the heightened mitochondrial or peroxisomal oxidative capacity observed in *Tspo* KO likely serves as a protective mechanism against TAG accumulation in the liver. So far, our findings suggest that the heightened transport of fatty acids from the plasma membrane in hepatocytes to mitochondria for FAO is facilitated by upregulated CD36, utilising the fatty acyl‐CoA‐Cpt1A pathway. This process appears to occur independently of the ACBP/DBI‐TSPO‐CPTA1 complex, as indicated by the increased transportation of fatty acids to the mitochondria for FAO in the absence of *Tspo*. Nevertheless, the absence of *Tspo* resulted in elevated levels of CPT1A, consequently boosting the transport of fatty acids to the mitochondria for FAO.

### 3.4 The Dysfunctional Mitochondria in *Tspo* KO Attributable to Impaired Mitochondrial Membrane Potential

**FIGURE 4:**
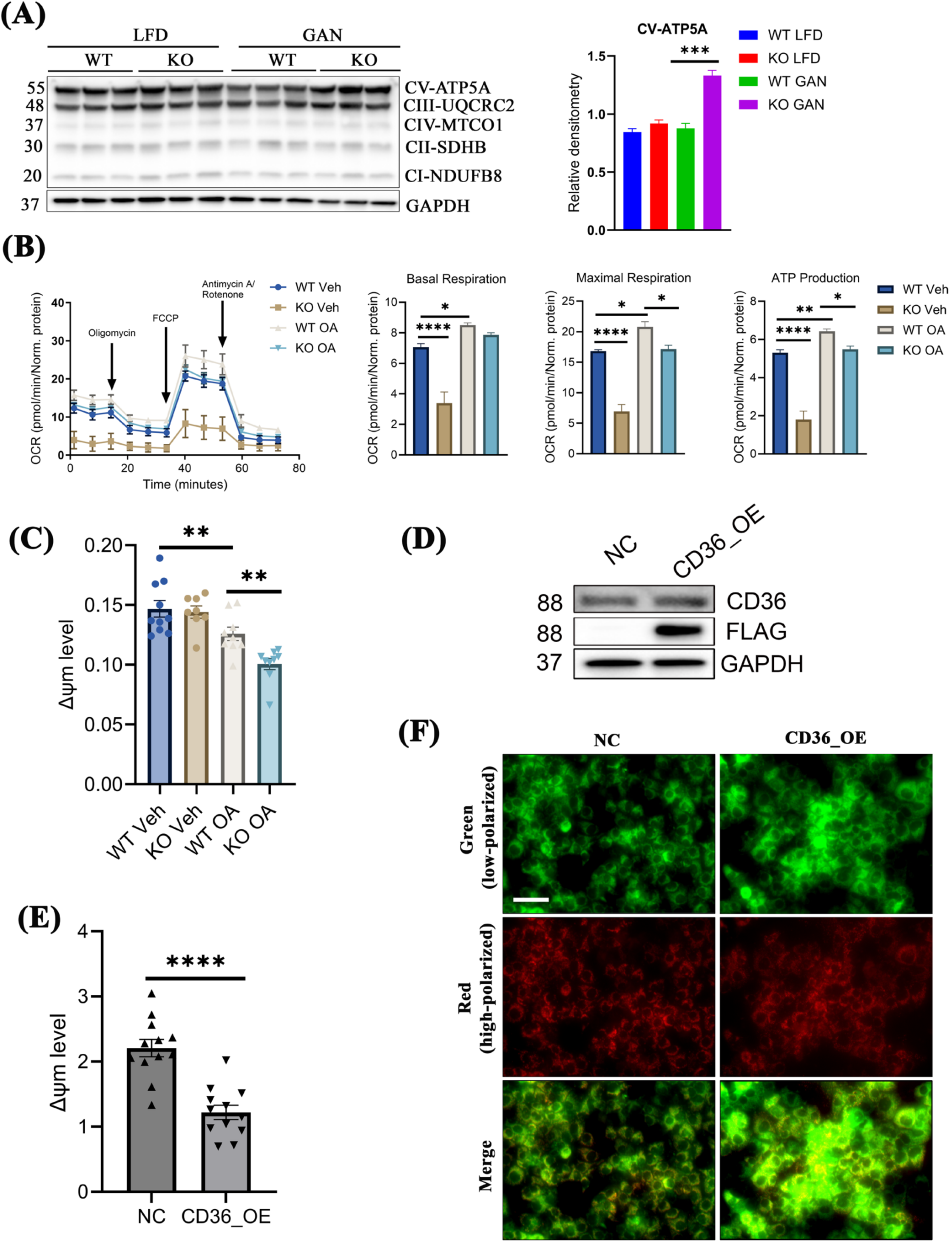
Malfunctional mitochondria in *Tspo* KO with GAN diet. (A) Immunoblot of OXPHOS comprising five complexes (complexes I–V [CI–CV]) in the liver tissue of rats and quantification (*n* = 3). (B) Seahorse assay in the cultured hepatocytes isolated from WT or KO rat livers and treated with BSA or 0.5 mM OA for 24 h. Quantification of oxygen consumption rate (OCR) for basal respiration, maximal respiration, and ATP production (*n* = 3). (C) Mitochondrial membrane potential (ΔΨm) test in cultured hepatocytes isolated from WT and KO rat livers treated with BSA or OA (*n* = 3). (D) Immunoblot analysis of CD36 and FLAG in Huh7 and transfected Huh7 with human CD36‐FLAG plasmid after 2 weeks of hygromycin (0.5 mg/mL) selection. NC: Huh7 negative control cells. CD36_OE: Stably overexpressed CD36 in Huh7. (E) Mitochondrial membrane potential (ΔΨm) test in NC (Huh7) and CD36_OE (*n* = 3). (F) Images for ΔΨm in NC (Huh7) and CD36_OE (*n* = 3). Green colour indicated low‐polarised signal; red colour indicated high‐polarised signal. ATP, adenosine triphosphate; BSA, bovine serum albumin; CD36‐FLAG, cluster of differentiation 36 with FLAG tag; OA, oleic acid; OXPHOS, oxidative phosphorylation. Scale bar: 100 μm. Data are presented as mean ± SEM, *p < 0.05, **p < 0.01, ***p < 0.001, ****p < 0.0001 by one‐way ANOVA.

Fatty acid oxidation is the metabolic process through which fatty acid molecules are broken down to produce acetyl‐CoA, nicotinamide adenine dinucleotide reduced (NADH), and reduced form of flavin adenine dinucleotide (FADH2), which then contribute to the electron transport chain (ETC) for adenosine triphosphate (ATP) synthesis [27, 28]. Consequently, we assessed the expression of the oxidative phosphorylation system (OXPHOS), comprising complexes I–V (CI‐CV) within the IMM, responsible for ATP production [29]. Immunoblot analysis revealed that levels of CV‐ATP5A exhibited minimal changes under an LFD but were significantly elevated in KO GAN compared to WT GAN (Figure 4A). There were no discernible differences in CI–CIV between WT and KO, irrespective of diet type (Figure 4A). The heightened IR and increased lipid accumulation observed in *Tspo* KO mice compared to WT with GAN diet suggest the adaptation or restructuring of mitochondrial energetics, recognised as a critical factor in the transition from SS to MASH [30]. Furthermore, we investigated whether the induced β‐oxidation and heightened OXPHOS observed in KO would impact ATP production. To this end, we isolated primary hepatocytes from WT and KO rats and exposed them to OA to assess their cellular bioenergetics. Results from the Seahorse XF Mito stress test indicated that OA‐treated primary WT hepatocytes exhibited increased oxygen consumption rates (OCR) at both basal and maximal respiration, along with heightened ATP production compared to basal, whereas these OCR significantly decreased in KO hepatocytes relative to WT upon OA treatment (Figure 4B). Subsequently, we investigated potential disruptions in mitochondrial membrane potential (MMP, Δψm). This was prompted by two primary considerations. Firstly, the absence of TSPO could potentially influence mitochondrial membrane integrity due to its transmembrane properties. Secondly, a decline in mitochondrial Δψm may lead to a reduction in ATP synthesis [31, 32]. We treated primary hepatocytes isolated from WT and *Tspo* KO rats with Veh or OA followed by MMP assay; the results showed that there was a significant decrease in MMP after OA treatment, indicating a compromised Δψm due to fatty acid stimulation. Particularly striking was the more pronounced reduction in MMP observed in the *Tspo* KO group compared to the WT following OA treatment (Figure 4C). Additionally, we assessed whether the elevation of CD36, a consequence associated with *Tspo* loss in MASH, could similarly influence MMP in hepatocytes. To do so, we established a Huh7 cell line with stably overexpressed CD36 (CD36_OE) evidenced by the immunoblot analysis of CD36‐FLAG expression only in CD36_OE (Figure 4D). This decrease was visualised by heightened low‐polarised mitochondrial signals in the CD36_OE group relative to the negative control (NC) group (Figure 4E,F).

### 3.5 RAPTOR Was Acetylated by Upregulated AcCoA After *Tspo* Loss in SS

**FIGURE 5:**
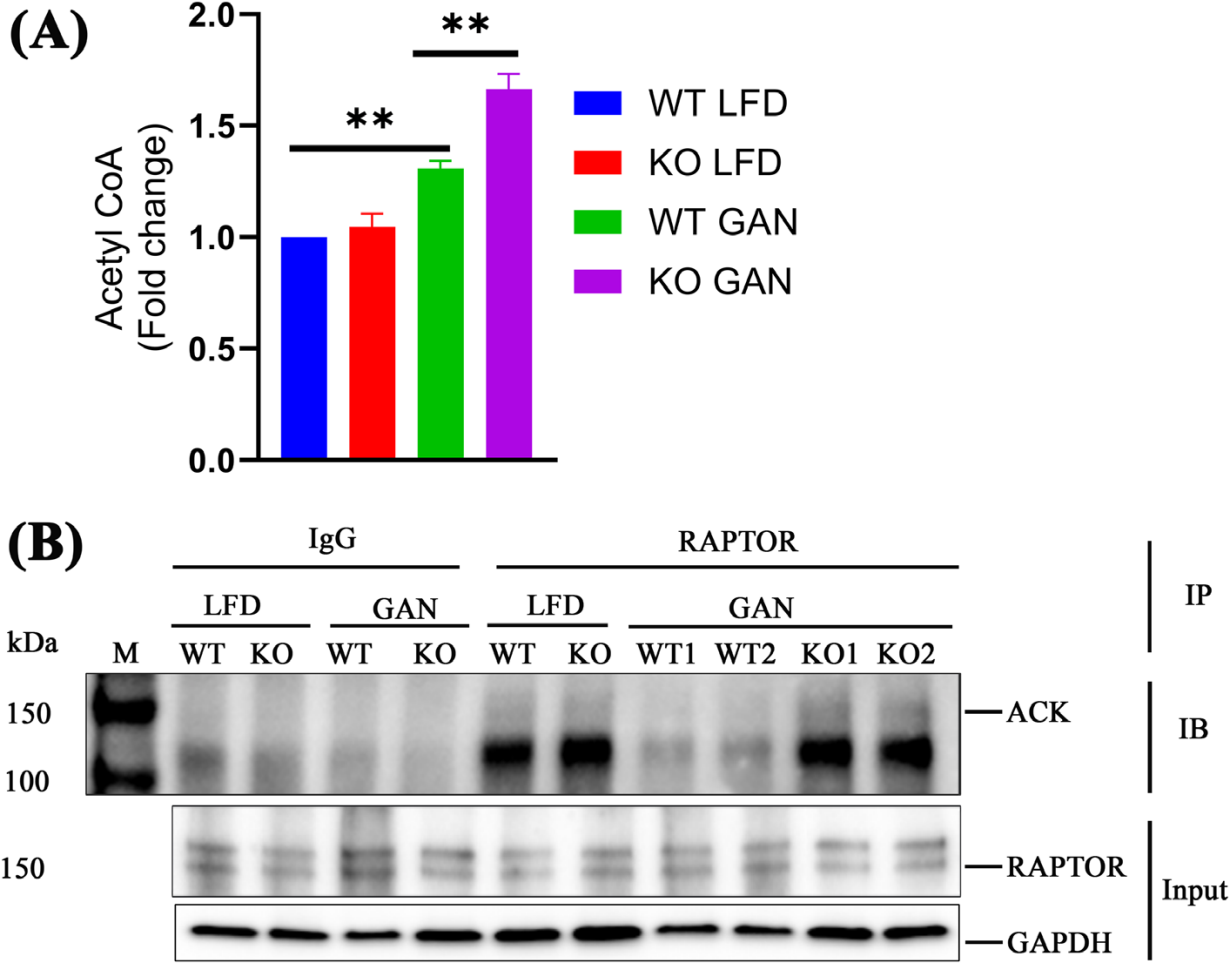
RAPTOR acetylation in KO GAN. (A) Measurement of Acetyl‐CoA (AcCoA) in liver tissues from rat (*n* = 6). (B) IP with IgG or RAPTOR antibody and IB using an antibody against acetylated‐lysine (ACK); IB of RAPTOR and GAPDH is input control. IB, immunoblot; IgG, immunoglobulin G; IP, immunoprecipitation; RAPTOR, regulatory‐associated protein of mTOR. Data are presented as mean ± SEM, **p < 0.01 by one‐way ANOVA.

In each cycle of β‐oxidation, one molecule each of AcCoA, NADH, and FADH_2_ is generated [28]. Notably, the heightened FAO observed in *Tspo* KO livers after the GAN diet implies an augmentation in AcCoA production. To delve deeper, we quantified total AcCoA levels in liver lysates. Our results unveiled a significant elevation in AcCoA levels in KO compared to WT rats following the GAN diet (Figure 5A). AcCoA functions as a versatile molecule, powering both energy production via the tricarboxylic acid (TCA) cycle and anabolic processes, such as lipid synthesis, within cells [33]. A recent study suggested that AcCoA could mediate autophagy through the acetylation of RAPTOR, a component of the mTORC1 complex. Subsequently, we aimed to ascertain whether the elevated levels of AcCoA impact downstream RAPTOR acetylation, a crucial event for RAPTOR binding to Ras‐related (Rag) GTPases on the lysosome and subsequent mTORC1 activation [34]. To investigate this, we conducted immunoprecipitation (IP) assays using RAPTOR or IgG antibodies with liver lysates, followed by immunoblot analysis using an antibody targeting acetylated lysine (ACK). The findings revealed heightened levels of RAPTOR acetylation in KO compared to WT after the GAN diet. Notably, no RAPTOR acetylation was detected in immunoblots using IgG for IP (Figure 5B), underscoring the specificity of RAPTOR acetylation.

### 3.6 Autophagy Impairment in *Tspo* KO Under a GAN Diet

**FIGURE 6:**
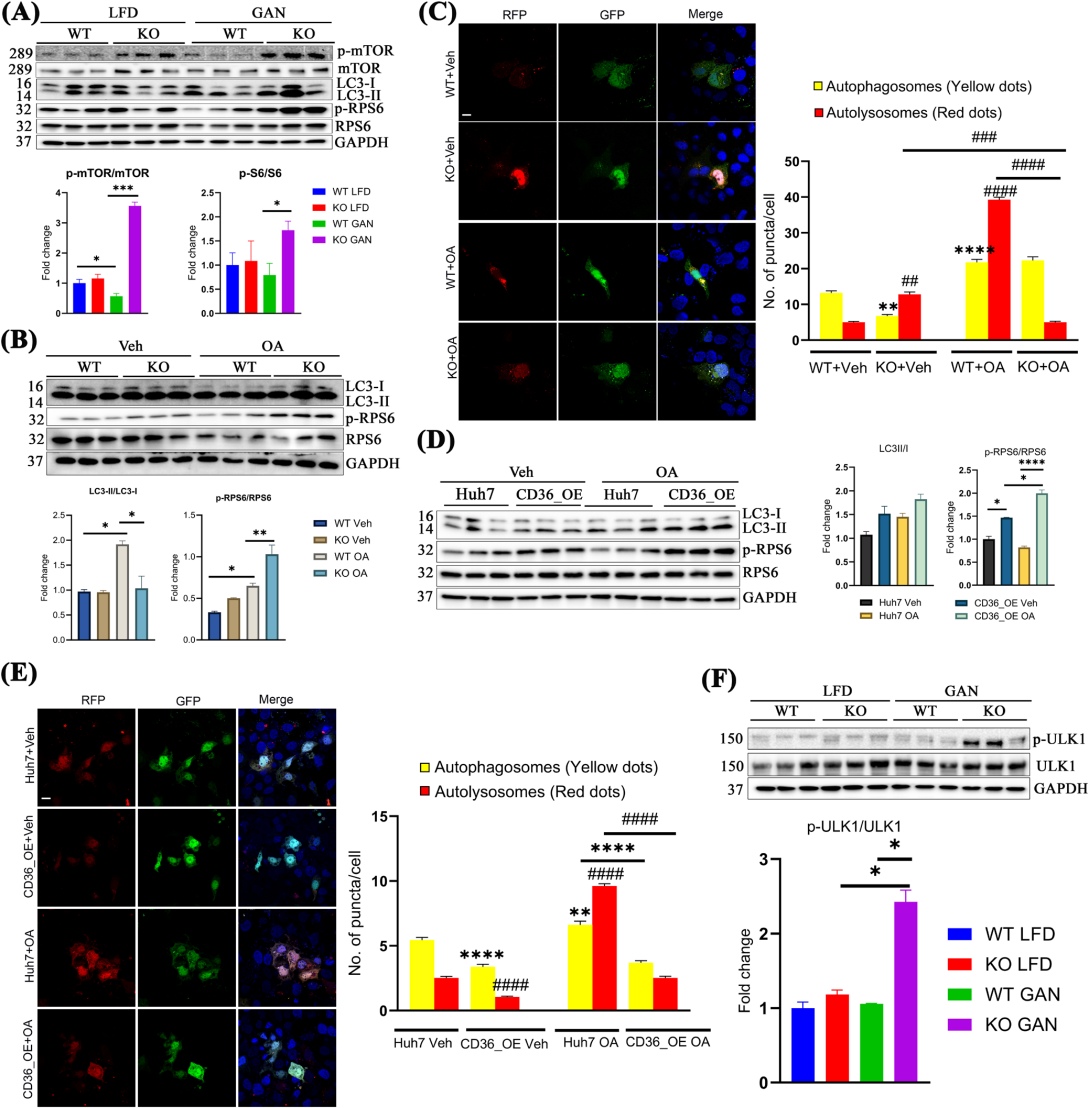
Autophagy impairment in *Tspo* KO with GAN diet. (A) Immunoblot analysis of p‐mTOR, mTOR, LC3, P62, p‐RPS6, and RPS6 in the liver tissues and quantification (*n* = 3). (B) Immunoblot analysis of LC3, p‐RPS6, RPS6 in cultured rat hepatocytes isolated from WT and KO treated with Vehicle (Veh) or OA (*n* = 3). (C) Representative confocal images in transiently transfected WT and KO rat primary hepatocytes with mRFP‐GFP‐LC3 plasmid followed by treatment with Veh or OA. Cells were fixed and imaged with 515/530‐nm band‐pass filter (green), or a 580‐nm long‐pass filter (red). Nuclei were stained with DAPI (blue). The bar graph shows quantification of yellow puncta (mRFP‐GFP‐LC3 positive) and red puncta (mRFP‐LC3 positive) per cell (*n* = 20 cells). Statistically significant changes compared to control cells (WT + Veh) are indicated with * (autophagosomes) or # (autolysosomes) above bars and statistically significant changes between the treated cells are indicated with * or # above brackets. (D) Immunoblot analysis of LC3, p‐RPS6, and RPS6 in Huh7 and CD36_OE Huh7 cells treated with Veh or OA and quantification (*n* = 3). (E) Representative confocal images in transiently transfected Huh7 cells or CD36_OE Huh7 cells with mRFP‐GFP‐LC3 plasmid followed by treatment with Veh or OA. Cells were fixed and imaged with 515/530‐nm band‐pass filter (green), or a 580‐nm long‐pass filter (red). Nucleus was stained with DAPI (blue). The bar graph shows quantification of yellow puncta (mRFP‐GFP‐LC3 positive) and red puncta (mRFP‐LC3 positive) per cell (*n* = 20 cells). Statistically significant changes compared to control cells (Huh7 Veh) are indicated with * (autophagosomes) or # (autolysosomes) above bars and statistically significant changes between the treated cells are indicated with * or # above brackets (*n* = 10 images per group). (F) Immunoblot analysis of p‐ULK1 (Ser757) and ULK1 in the liver tissue (*n* = 3). Scale bar: 20 μm. mTOR: Mechanistic target of rapamycin; LC3: Microtubule‐associated protein 1A/1B‐light chain 3; P62: Sequestosome 1 (SQSTM1); RPS6: Ribosomal protein s6; OA: Oleic acid; mRFP‐GFP‐LC3: Microtubule‐associated protein 1A/1B light chain 3B (LC3) fused with mRFP and GFP. Data are presented as mean ± SEM, * (or #) *p* < 0.05, ** (or ##) *p* < 0.01, *** (or ###) *p* < 0.001, **** (or ####) *p* < 0.0001 by one‐way ANOVA analysis.

If AcCoA in KO GAN acetylates RAPTOR to activate mTORC (p‐mTORC), a major negative regulator of endosomal biogenesis and autophagy, then there should be impairment in the autophagy flux in KO GAN. To investigate this, we first confirmed higher p‐mTOR/mTOR levels in KO GAN than in WT GAN (Figure 6A). Immunoblot analysis also showed an increase in the LC3II/LC3I ratio after GAN diet in WT rats, but a lower increase in KO rats on the GAN diet (WT LFD: 1.00 ± 0.31, KO LFD: 1.20 ± 0.29, WT GAN: 2.81 ± 0.62, and KO GAN: 2.39 ± 0.12). The heightened levels of the sequestosome marker P62 in conjunction with increased LC3‐II/LC3I ratios suggest impaired degradation of autophagosomes at later stages of autophagic processing. Furthermore, the levels of ribosomal protein S6 phosphorylation (p‐RPS6), a commonly acknowledged marker of mTORC1 activity associated with autophagy inhibition [35], exhibited a substantial increase in KO GAN liver samples compared to WT GAN counterparts (Figure 6A). These data suggest that autophagy progression was impaired not only at the initiation stage but also during autophagosome degradation.

We next treated primary hepatocytes isolated from WT and KO rats with OA. OA treatment induced autophagy, as evidenced by a significant upregulation of the LC3II/LC3I ratio, which mimics the induction of autophagy observed in vivo. Compared to WT OA, the LC3II/LC3I was reduced, but p‐RPS6/RPS6 was significantly increased (Figure 6B), indicating impaired autophagy in KO animals with OA treatment. To visualise if autophagic degradation was prevented in KO hepatocytes after OA treatment, we transfected primary hepatocytes from WT and KO rats with a plasmid expressing mRFP‐GFP tandem fluorescent‐tagged LC3 (tfLC3) following OA treatment (Figure S4). Under normal conditions, autophagosomes display both GFP and mRFP fluorescence signals (yellow dots), whereas autolysosomes primarily exhibit only mRFP fluorescence (red dots) because the green GFP fluorescence is quenched in the acidic lysosomal environment (pH 4.5–5.5). Thus, an increased number of yellow dots indicates impaired autophagic flux, whereas an increased number of red dots indicates enhanced autophagic processing. After OA treatment, red dots were abundant in WT hepatocytes, suggesting a substantial increase in autolysosome formation. However, red dots were significantly reduced in OA KO hepatocytes compared to vehicle‐treated KO cells, indicating that *Tspo* deficiency hampers autophagic progression (Figure 6C).

To check whether overexpression of CD36 in Huh7 cells can simulate *Tspo* KO after lipid loading, we treated Huh7 and CD36_OE cells with OA. Immunoblot analysis revealed significant increases in p‐RPS6/RPS6 but not much change in LC3‐II/LC3‐I in CD36_OE cells compared to Huh7 cells following OA treatment (Figure 6D). This impairment in the autophagic process was evidenced by a decrease in the number of autolysosomes (red dots) in CD36_OE OA cells compared to Huh7 OA cells (Figure 6E).

Unc‐51‐like kinase 1 (ULK1) is a serine/threonine kinase that participates in the initiation of autophagy [36]. We therefore examined changes in ULK1 in vivo. Immunoblot analysis revealed that the ULK1 level was suppressed by phosphorylation at Ser757 in KO GAN (LFD WT: LFD KO: GAN WT: GAN KO = 1:1:1:4.8) (Figure 6F), suggesting that autophagy initiation was impaired in *Tspo* KO with GAN diet compared to that in WT GAN.

## 4 Discussion

**FIGURE 7:**
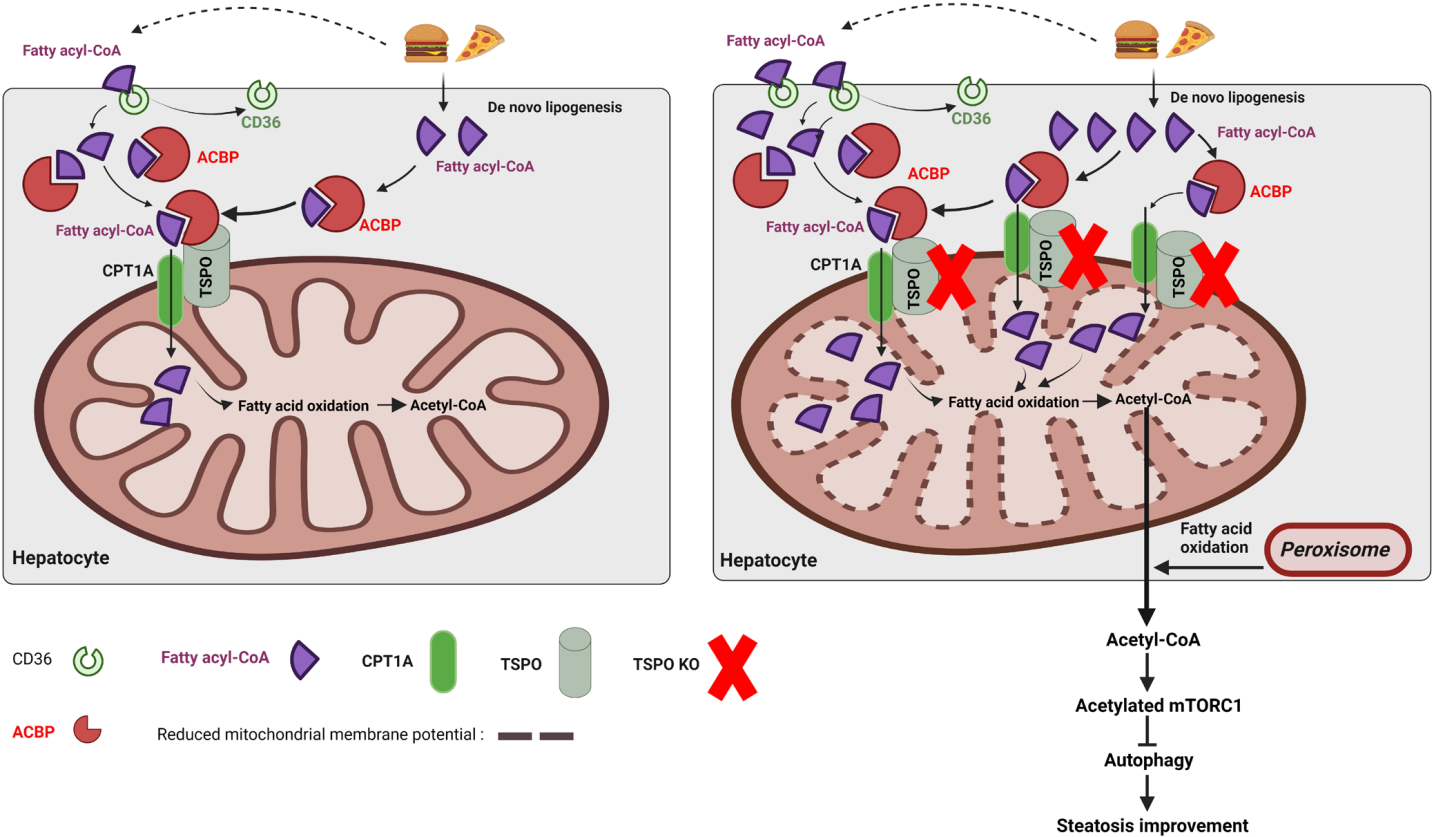
*Tspo* KO exacerbates hepatic steatosis. Left panel: GAN leads to simple steatosis in WT rats. Right panel: *Tspo* KO exacerbates hepatic steatosis by upregulating CD36, which promotes the uptake of free fatty acids (FFA) and enhances de novo lipogenesis (DNL). The elevated FFA levels are transported into mitochondria through binding to CPT1A, facilitating fatty acid oxidation (FAO) and generating Acetyl‐CoA (AcCoA). The upregulated AcCoA acetylates mTORC1, impairing autophagic degradation. Furthermore, the upregulation of CD36 leads to dysfunctional mitochondria characterised by compromised mitochondrial membrane potential. Consequently, these cascading mechanisms lead to severe hepatic steatosis in *Tspo* KO liver. ACBP, acyl‐CoA binding protein; CD36, cluster of differentiation 36; CPT1A, carnitine palmitoyltransferase 1a; GAN, Gubra‐Amylin NASH; mTORC1, mechanistic target of rapamycin complex 1.

The rats lacking *Tspo* developed insulin resistance (IR) at an early stage of MASLD. DNL and CD36‐mediated FA uptake contribute to lipid accumulation in *Tspo* KO rats fed a GAN diet for 8 weeks. TSPO interacts with CPT1A in the OMM. While FA transport to mitochondria for FAO primarily relies on CPT1A rather than TSPO, *Tspo* deficiency leads to an increase in CPT1A expression, enhancing FAO. However, mitochondrial function is impaired in *Tspo* KO rats after lipid loading, likely due to impaired MMP, observed in both *Tspo* KO hepatocytes and CD36‐overexpressing Huh7 cells. Additionally, loss of *Tspo* results in the upregulation of AcCoA in response to the GAN diet, which activates mTORC1 via acetylated RAPTOR, thereby inhibiting autophagy. This finding was validated in primary *Tspo* KO hepatocytes and CD36‐overexpressing Huh7 cells. The proposed working model, illustrated in Figure 7, suggests that TSPO orchestrates the regulation of lipid metabolism‐mediated autophagic degradation during the early stages of MASLD.

Conflicting results regarding changes in autophagy in MASLD patients have been reported by Gonzalez‐Rodriguez [35] and Lou [37]. In a study involving a high‐fat diet (HFD)‐induced mouse model of MASLD, hepatic autophagy was found to be enhanced at 4, 8, 12, and 16 weeks of treatment [38]. In our current study, we observed upregulation of autophagy after 8 weeks of a GAN diet, as evidenced by increased LC3‐II/I levels and decreased P62 and p‐RPS/RPS. Since OA treatment in vitro induced autophagy, we also used OA treatment for our in vitro study.

IR in MASLD manifests as reduced sensitivity to insulin throughout the body, including hepatic and adipose tissues [39]. IR contributes significantly to fat accumulation and deposition in MASLD, driven by various mechanisms such as excessive dietary fat intake, increased delivery of FFAs to the liver, and heightened DNL [10, 11]. IR exacerbates hepatic fat accumulation by promoting FFA delivery to the liver and enhancing the anabolic effects of hyperinsulinaemia [40]. Previous studies have shown that patients with both SS and IR exhibit elevated hepatic mitochondrial FAO and respiratory function either in vivo or ex vivo following mitochondrial isolation from liver biopsies [33, 41]. However, as hepatocytes accumulate more lipids than their storage capacity, excessive fatty acid uptake starts impairing mitochondrial function [42]. During the progression from SS to MASH, hepatic mitochondrial respiration declines while reactive oxygen species (ROS) production increases. This adaptation of hepatic mitochondrial function observed in patients with SS is lost in those with steatohepatitis [33]. In our study, we observed developed IR in the *Tspo* KO GAN group rats, leading to increased DNL and FFA uptake, without changes in VLDL‐TAG secretion. Consequently, *Tspo* KO rats showed heightened FAO with the GAN diet, indicated by the upregulation of CPT1A, PPARα, and ACOX1, key enzymes responsible for FAO. However, our in vitro study using isolated *Tspo* KO cells treated with OA suggested compromised mitochondrial functions. This investigation implies that *Tspo* loss triggers early‐stage steatosis in MASLD due to mitochondrial dysfunction.

Through screening for potential mechanisms explaining why TSPO affects lipid accumulation, we discovered that CD36 acts as a mediator linking TSPO to the transport of lipids from the plasma membrane of hepatocytes to the mitochondria. CD36 facilitates the transport of long‐chain fatty acids [43]. Studies have shown that overexpression of *Cd36* enhances hepatic fatty acid uptake and fat accumulation, whereas liver‐specific knockout of *Cd36* reduces hepatic lipid levels in both genetically induced and diet‐induced steatosis [44]. These findings strongly suggest a causal role of CD36 in steatosis, which is further supported by abnormally increased CD36 levels observed in patients with MASLD. To validate these findings in vivo, overexpressed CD36 in hepatocytes mimicked the effects of *Tspo* loss in primary hepatocytes treated with OA. Importantly, overexpression of *CD36* in human hepatocytes or *Tspo* knockout in primary hepatocytes treated with OA impaired MMP. This observation may help explain why FAO increases but overall mitochondrial function decreases in the early stages of MASLD due to *Tspo* loss.

Furthermore, we provided evidence demonstrating the causal relationship between CD36 overexpression and the loss of *Tspo*, which is associated with autophagy, likely through the upregulation of AcCoA levels. FAO‐derived AcCoA will either go to TCA for more ATP production or will be the substrate for DNL [45, 46]. However, we found that when ATP production was reduced, even higher FAO occurred with T *Tspo* KO. This also explains AcCoA‐mediated DNL upregulation in *Tspo* KO animals. Importantly, we provided additional evidence indicating that the increased AcCoA levels not only contribute to the functions mentioned above but also acetylate RAPTOR to activate mTORC1. This establishes a connection between *Tspo* loss, CD36 overexpression, and the impairment of autophagy in hepatic steatosis. Our results are not only in agreement with earlier studies that impaired autophagy is linked to the initial development of hepatic steatosis and the progression of steatosis to liver injury [47, 48], but underscore the indispensable role of TSPO in maintaining hepatic lipid metabolism. Serendipitously, our previous study in steroidogenic MA‐10 *Tspo* WT cells revealed that upon hormonal stimulation, mitochondria efficiently import cholesterol for steroid production at the expense of other lipids necessary for energy production, specifically fatty acids β‐oxidation [49]. This indicates a balance between cholesterol uptake and fatty acid utilisation for metabolic homeostasis. In this study, we demonstrated that the loss of *Tspo* disrupted this balance between cholesterol uptake and fatty acid β‐oxidation, eventually leading to lipid accumulation following a GAN diet. Collectively, our data provide mechanistic insights into how TSPO influences DNL, CD36‐mediated fatty acid transport, and autophagy inhibition, all contributing to the progression of SS to later stages.

## Author Contributions

**Yuchang Li:** conceptualization (equal), data curation (equal), formal analysis (lead), investigation (lead), methodology (equal), writing – original draft (equal), writing – review and editing (equal). **Liting Chen:** data curation (supporting), formal analysis (equal), investigation (supporting), methodology (equal), writing – original draft (supporting), writing – review and editing (supporting). **Chantal Sottas:** investigation (supporting), methodology (supporting), writing – original draft (supporting), writing – review and editing (supporting). **Nrupa Dinesh Patel:** investigation (supporting), methodology (supporting), writing – original draft (supporting), writing – review and editing (supporting). **Mahima Chandrakant Raul:** investigation (supporting), methodology (supporting), writing – original draft (supporting), writing – review and editing (supporting). **Vassilios Papadopoulos:** conceptualization (equal), funding acquisition (lead), investigation (equal), project administration (lead), resources (lead), supervision (lead), writing – original draft (equal), writing – review and editing (equal).

## Conflicts of Interest

The authors declare no conflicts of interest.

## Supporting information


**Supplementary Files**.

Data S1.

## Data Availability

All data generated are presented in the manuscript.
