# Supplementary material for: Tspo Depletion Exacerbates Steatosis Through Fatty Acid Uptake
Source: J Cell Mol Med. 2025 Apr 7;29(7):e70500. doi: 10.1111/jcmm.70500 (PMC11975627; doi:10.1111/jcmm.70500)
Supplement: Supplementary file 1 — Supplementary Files. [file JCMM-29-e70500-s001.docx]

**Supplementary Information**

# *Tspo* Depletion Exacerbates Steatosis through Fatty Acid Uptake

Yuchang Li, Liting Chen, Chantal Sottas, Nrupa Dinesh Patel, Mahima Chandrakant Raul, and Vassilios Papadopoulos*

Department of Pharmacology and Pharmaceutical Sciences, Alfred E. Mann School of Pharmacy and Pharmaceutical Sciences, University of Southern California, Los Angeles, CA 90089, USA

**Figure S1.** Food intake in WT and *Tspo* KO rats during 8-week LFD or GAN diet feeding. Food intake of WT and *Tspo* KO rats fed either an LFD or GAN diet for 8-week (n=10). Data are presented as mean ± SEM; one-way ANOVA was used for statistical analysis, LFD: low fat diet; GAN: Gubra-Amylin NASH; ns: no significance. Note: Food intake was measured daily during the feeding period.


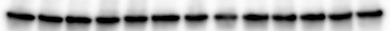

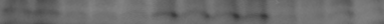


TSPO

37

GAPDH

LFD

WT

KO

GAN

WT

KO

20

kDa

**Figure S2.** Immunoblot analysis of TSPO and GAPDH in WT LFD (n=2), *Tspo* KO LFD (n=3), WT GAN (n=4), and *Tspo* KO GAN (n=3). LFD: low fat diet; GAN: Gubra-Amylin NASH.


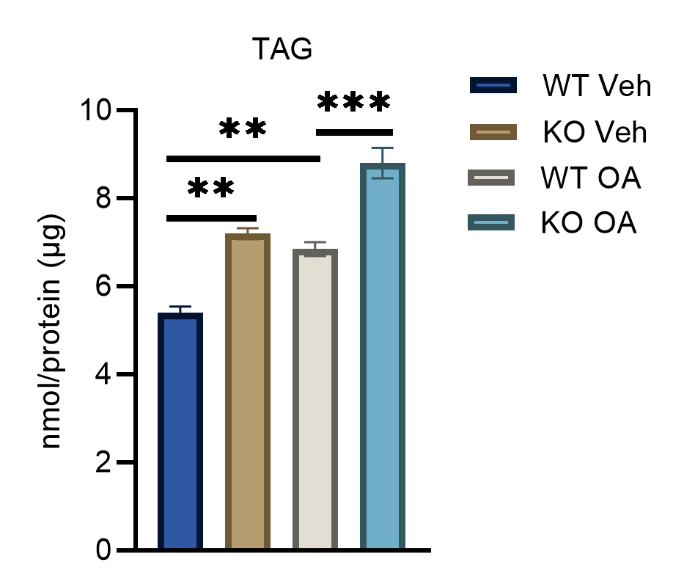


**Figure S3**. TAG measurement in the primary hepatocytes isolated from WT and *Tspo* KO rats (n=3). The primary cells were treated with either BSA (Veh) or 0.5mM OA for 24hrs. Then the cells were pelleted for TAG measurement. Data are presented as mean ± SEM, ∗∗p < 0.01, ∗∗∗p < 0.001 by one-way ANOVA. TAG: triacylglycerol; BSA: bovine serum albumin; OA: oleic acid.


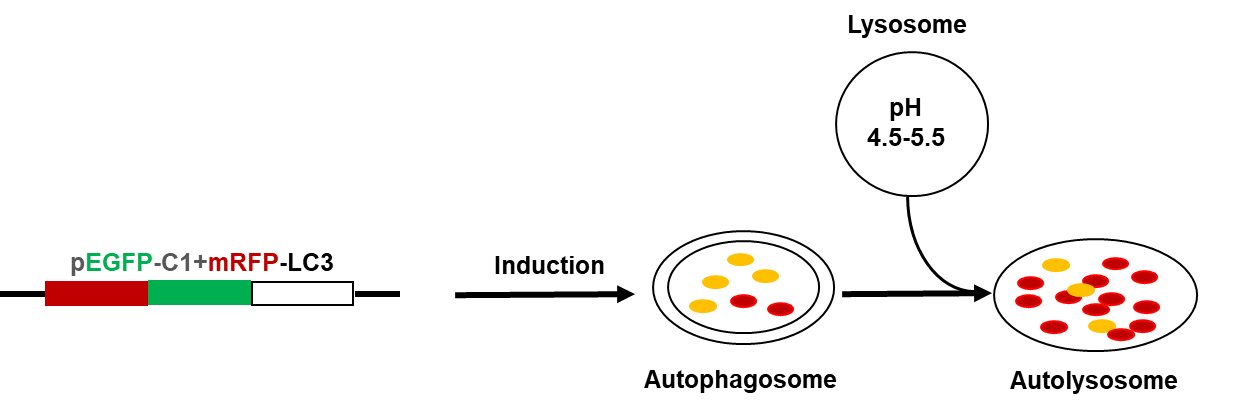


**Figure S4**. Schematic diagram of the pGFP-C1+mRFP-LC3 reporter to measure autophagic flux.  pGFP-C1+mRFP-LC3 plasmid was transfected into the mammalian cell lines as indicated in the main text. When autophagy was induced, the cargo was engulfed by the phagophores and autophagosomes were formed. LC3 was lipidated and incorporated into autophagosomal membranes by the interaction of LC3 with the cargo receptors. In autophagosomes, GFP and RFP were emitted to demonstrate overlapping yellow fluorescence due to the basic environment.  When autophagy flux continued, autophagosomes fused with lysosomes to form autolysosomes. pGFP‑C1 green fluorescence was quenched due to lysosomal acidity (pH4.5-5.5), but mRFP‑LC3 was not and thus continued to show red fluorescence, indicative of the autophagic degradation process. pGFP-C1+mRFP-LC3: microtubule-associated protein 1A/1B-light chain 3 (LC3).

**Table S1.** Primers sequences used in the study in *Rattus Norvegicus* (rat) (Related to main Figures 2).

| GENE | FORWARD 5’ to 3’ | REVERSE 5’ to 3’ |
| --- | --- | --- |
| *Rps18* | ATAGCCAGGTTCTGGCCAAC | TTGGACACACCCACAGTACG |
| *Srebp-1c* | TCTTGACCGACATCGAAGACAT | CCAGCATAGGGGGCATCAAA |
| *Cd36* | GCTGATTACTTCTGTGTAGTAGCTT | GCTCCAGTAATGAGCCCACA |
| *Fatp1* | AGACTTCTGCGAGAACCCGT | TCAGAACAGAGAGGCCAAAGAG |
| *Fatp5* | TGTAATGTCCCAGGGCAACC | TCAAGGGGAGTCAGCATTCG |
| *Mttp* | CGAGTGAAAAATCGGGTGGC | TCGGCTTTGTCCATCTGCAT |

**Table S2.** List of antibodies used in immunoblot (Related to main Figures 1-6).

| **Primary antibodies** | **Supplier** | **Reference** | **Dilution** |
| --- | --- | --- | --- |
| CPT1A | Abcam | #ab128568, RRID: AB_11141632 | 1:1000 |
| PPAR alpha | Abcam | # ab24509, RRID: AB_448110 | 1:1000 |
| OXPHOS | Abcam | # ab110413, RRID: AB_2629281 | 1:1000 |
| ACBP/DBI | Abclonal | #A13293, RRID: AB_2760152 | 1:1000 |
| Acetylated lysine (AcK) | Cell signaling | #9681, RRID: AB_331799 | 1:1000 |
| FASN | Cell signaling | #3180, RRID: AB_2100796 | 1:1000 |
| GAPDH | Cell signaling | # 2118, RRID: AB_561053 | 1:10000 |
| LC3B | Cell signaling | #2775, RRID: AB_915950 | 1:2000 |
| p-mTOR | Cell signaling | # 2971, RRID: AB_330970 | 1:1000 |
| mTOR | Cell signaling | # 2983, RRID: AB_2105622 | 1:1000 |
| p-RPS6 | Cell signaling | # 2215, RRID: AB_331682 | 1:1000 |
| RPS6 | Cell signaling | # 2217, RRID: AB_331355 | 1:1000 |
| MYC-Tag | Cell signaling | # 2278, RRID: AB_490778 | 1:1000 |
| p-ULK1(Ser757) | Cell signaling | #14202, RRID: AB_2665508 | 1:1000 |
| Rabbit IgG | Novus Biologicals | #NB810-56910, RRID: AB_844243 | 1:1000 |
| TSPO | ProSci Inc | Generated by Dr. Papadopoulos lab | 1:1000 |
| ACOX1 | Proteintech | # 10957-1-AP, RRID: AB_2221670 | 1:1000 |
| DYKDDDDK (FLAG) | Proteintech | #20543-1-AP, RRID: AB_11232216 | 1:1000 |
| RAPTOR | Proteintech | # 20984-1-AP, RRID: AB_11182390 | 1:1000 |
| TNF Alpha | Proteintech | # 17590-1-AP, RRID: AB_2271853 | 1:1000 |
| ULK1 | Proteintech | # 20986-1-AP, RRID: AB_2878783 | 1:1000 |
| ACTA2 | Sigma | #A2547, RRID: AB 476701 | 1:1000 |
| CD36 | SinoBiological | # 80263-T48 | 1:1000 |
|  |  |  |  |
| **Secondary antibodies** | **Supplier** | **Reference** | **Dilution** |
| Anti-Mouse HRP | Li-Cor | # 926-80010: RRID AB_2721263 | 1:5000 |
| Anti-Rabbit HRP | Li-Cor | # 926-80011, RRID: AB_2721264 | 1:5000 |

**Table S3.** Antibodies for immunohistochemistry staining or proximity ligation assay (Related to main Figure 2, 3).

| **PRIMARY ANTIBODIES** | **SUPPLIER** | **REFERENCE** | **DILUTION** |
| --- | --- | --- | --- |
| CPT1A | Abcam | #ab128568, RRID: AB_11141632 | 1:400 |
| CD36 | Novus Biologicals | # NB400-144, RRID: AB_10003498) | 1:400 |
| TSPO | ProSci Inc | Generated by Dr. Papadopoulos lab | 1:400 |
| TSPO | Proteintech | # 68137-1-Ig, #RRID: AB_2923664 | 1:400 |
| ACBP/DBI | Abclonal | #A13293, RRID: AB_2760152 | 1:400 |
