# Supplementary material for: Tspo Depletion Exacerbates Steatosis Through Fatty Acid Uptake
Source: J Cell Mol Med. 2025 Apr 7;29(7):e70500. doi: 10.1111/jcmm.70500 (PMC11975627; doi:10.1111/jcmm.70500)
Supplement: Supplementary file 2 — Data S1. [file JCMM-29-e70500-s002.docx]

## **Materials and Methods**

### Intraperitoneal insulin tolerance test (ITT) and intraperitoneal glucose tolerance test (GTT)

At the 6th week of feeding, insulin tolerance tests (ITT) and glucose tolerance tests (GTT) were conducted. For ITT, rats were fasted for 6 hours, followed by an intraperitoneal injection of insulin (0.75 IU/kg, #NDC0002-7510-17, Humalog, Lilly). For GTT, rats underwent a similar 6-hour fast, followed by an intraperitoneal injection of 2 g/kg glucose (#NDC 13985-067-00, VETone). Subsequently, the rats were anesthetized with isoflurane (NDC 13985-528-60) for 2-3minutes in the Tabletop Laboratory Animal Anesthesia System (#901806, VETEQUIP, USA), and blood samples were collected from the tip of the tail vein at 0, 0.5, 1-, and 2-hours post-injection. Blood glucose levels were immediately measured using the FreeStyle Lite blood glucose strips and monitoring system (NDC 99073-0708-05, FreeStyle).

### In Vivo VLDL-Triacylglycerol secretion

At the 7th week of feeding, VLDL-Triacylglycerol secretion was assessed by intraperitoneal injection of 1 mg/kg Tyloxapol BioXtra (#T0307, Sigma-Aldrich) in sterile saline after a 4-hour fasting period. The rats were anesthetized with isoflurane (NDC 13985-528-60) for 2-3minutes in the Tabletop Laboratory Animal Anesthesia System (#901806, VETEQUIP, USA), and sequential blood samples were collected from the tip of the tail vein at 0-, 1-, and 2-hours post-injection. Blood samples were then centrifuged at 1,000 g for 10 minutes at 4°C. The resulting serum samples were transferred to a new tube and sent to IDEXX Laboratories, Inc. (Westbrook, ME) for analysis of the TAG levels.

### Plasma testing

After 8 weeks of feeding, rats were anesthetized with isoflurane (NDC 13985-528-60) for 2-3minutes in the Tabletop Laboratory Animal Anesthesia System (#901806, VETEQUIP, USA), and subsequently decapitated. Trunk blood was collected into microcontainers (#365965, BD Microtainer Blood Collection Tubes) and centrifuged at 1200g for 10 minutes to harvest plasma. The levels of alanine aminotransferase (ALT), aspartate aminotransferase (AST), total cholesterol, and triacylglycerol (TAG) were measured by Antech Diagnostics (Fountain Valley, CA, USA).

### Hepatic triacylglycerol (TAG) quantification

The frozen liver tissue was cut into small pieces (5-15mg/sample) on dry ice and transferred to a new TissueLyzer tube (#990381, Qiagen). The liver tissue was homogenized in 500ul of 5% NP-40 solution with TissueLyzer II (Qiagen, USA) at 30 times/sec for 1 min. The cell lysate was used for TAG measurement according to the instructions of the TAG quantification kit (#ab65336, Abcam). TAG contents were normalized to tissue weight (nmol/mg liver).

### Hematoxylin and eosin (H&E) staining

The livers were sliced with a scalpel to approximately 0.5 cm in maximum dimension and fixed in 4% paraformaldehyde (PFA) for 2 hours. They were then transferred to 70% ethanol at room temperature for overnight. Subsequently, the livers were processed for paraffin embedding. For H&E staining, the paraffin-embedded liver sections were incubated at 65°C for 30 min to soften the paraffin, then washed twice with xylene for 5 min, and rehydrated with an ethanol gradient series from 100%, 95%, 70% to 50% and finally rinsed with dH2O. Following a standard protocol, the deparaffinized liver sections were stained with H&E.(1). Images were acquired at 20x magnification using a NIKON 80i LIGHT MICROSCOPE with brightfield condensers. 10 random images were captured from each sample.

### Sirius red staining

Paraffin-embedded liver sections, prepared as described above for H&E staining, were incubated at 65°C for 30 min to soften the paraffin. The sections were then washed twice with xylene for 5 min each and rehydrated through a series of ethanol gradients (100%, 95%, 70%, 50%) and finally rinsed with dH_2_O. Slides were stained with Picro-Sirius Red Stain Kit (#KTPSRPT, American MasterTech), according to the manufacturer’s instructions. Briefly, the slides were immersed in Picro-Sirius red stain solution for 1min, followed by a 0.5% acetic acid rinse. After dehydration in absolute alcohol, the slides were cleared in xylene and mounted with permanent mounting media (#NB316, INNOVEX). The images were visualized and captured under a NIKON 80i LIGHT MICROSCOPE. The collagen density was quantified using Image J software.

### Oil Red O (ORO) staining

For ORO staining, the fresh liver tissue, approximately 0.5 cm in maximum dimension, was fixed with 4% PFA for 4 hours, followed by incubation with 15% sucrose in PBS for an additional 4 hours at 4°C. After incubation with 30% sucrose in PBS overnight, the tissues were cryo-embedded in Tissue Freeze Medium (#27209, Ted Pella, Inc. USA). The cryosections were cut at 10 μm in a cryostat (CM1900; Leica). For the ORO staining of the cryosections, the frozen sections were dried in air for 30 minutes. The slides were then post-fixed in 10% neutral buffered formalin for 10 min. After a quick dip in 60% isopropanol, the slides were stained in working ORO solution for 15 min and again dipped in 60% isopropanol for 1 min followed by deionized water for 1 min. Next, the slides were counterstained with Hematoxylin for 15 sec and dipped in water 10 times. The slides were then covered with aqueous mounting gel and observed under microscopy for analysis.

For ORO staining of cells, the cells on the coverslip (0.4M/ml) were fixed with 4% PFA for 10 min followed by PBS wash.  The cells were stained with ORO solution (#O1391, Millipore-Sigma) for 15 min followed by rinsing 3 times with dH20. The slides were then covered with aqueous mounting gel and observed under microscopy for analysis.

### Primary hepatocytes isolation

Primary rat hepatocytes were isolated using a two-step collagenase perfusion protocol through the portal vein (2). In brief, following anesthesia with a ketamine injection (Covetrus North America, catalog number: NDC11695-0703-1), the inferior vena cava was cannulated, and the portal vein was cut. The liver was then perfused with Hank’s Balanced Salt Solution (HBSS, #14025092, ThermoFisher) followed by a collagenase IV solution (1 mg/mL, #5138, Sigma) through the superior vena cava to break down the extracellular matrix and release the hepatocytes. The liver tissue was then gently disrupted to create a cell suspension, ensuring minimal damage to the cells. The cell suspension was centrifuged at 50 g for 2 minutes to pellet the hepatocytes. The pellet was resuspended in a 25% Percoll solution (#P4937, Sigma) and centrifuged again at 600 g for 15 minutes. After isolation, the cells over 93% viability were used for subsequent experiments. Upon isolation, the hepatocytes were cultured in Poly-D-Lysine Solution (#5049, Advanced Biomatrix) coated plates with basic DMEM medium supplemented with insulin (1µg/ml) and hydrocortisone (0.25µM) for 4 hrs. The medium was replaced with 10% Hi-FBS DMEM for overnight culture. The cells were used for experiments.

### Quantitative real-time PCR (qPCR)

Total RNA was extracted from cells or tissue using Quick-RNA™ MiniPrep (#R1054&1055, Zymo Research, USA). Reverse-transcription was performed using a PrimeScript RT reagent kit (#RR037A, Takara). qPCR was amplified with SYBR Green Real-Time PCR Master Mixes (#A25742, ThermoFisher) and performed on a CFX96 Touch Real-Time PCR Detection System (BIO-RAD). *Rps18* was used as internal reference. The primer sequences are listed in **Table S1**. The samples were run independently in triplicate.

### Co-immunoprecipitation (Co-IP)

For the TSPO-CPT1A interaction study. PBR (TSPO) (NM_000714) Human Tagged ORF Clone (TSPO-MYC-DDK plasmid) (#RC220107, OriGene) was transfected into HEK293 cells (#CRL-1573, ATCC), which were maintained in DMEM (#11965118, ThermoFisher) supplemented with 10% Hi-FBS (#F4135, Sigma) and 1% penicillin-streptomycin. The transfection process was carried out according to the instructions provided for the Lipofectamine 3000 Transfection Reagent (L3000008, ThermoFisher). After 24 hours of transfection, the cells were harvested and lyzed with lysis/equilibration buffer (#635721, Takara). The supernatants were collected after centrifugation at 17,000×g for 10 minutes at 4°C. The protein levels were determined using a Pierce Bradford Protein Assay Kit (#23225, ThermoFisher). Co-immunoprecipitation was performed per instruction of Capturem IP & Co-IP Kit (#635721, Takara) Myc-Trap agarose beads (catalogue # yta-20, Proteintech) was used according to the manufacturer’s specifications with minor modifications: 20µL beads were used per tube; following the final wash/spin step, complexes were eluted in 2X SDS sample buffer (125mM Tris-HCl [pH 6.8], 4% SDS, 20% glycerol, bromophenol blue) by boiling at 95°C for 10 minutes. Eluate was centrifuged at 2500*xg* for 5 min at 4°C and samples were stored at −70°C until use in immunoblotting.

For the Acetylated Raptor study, the liver lysate samples (2 WT LFD, 2 KO LFD, 3 WT GAN, and 3 GAN KO) were extracted after lysis/equilibration buffer (#635721, Takara) treatment followed by centrifugation at 17,000×g for 10 min at 4°C. The protein levels were determined using a Pierce Bradford Protein Assay Kit (#23225, ThermoFisher). Then 500 μg protein from individual liver samples (2 WT LFD, 2 KO LFD, 3 WT GAN, and 3 GAN KO) was incubated with an appropriate amount of IgG (#NB810-56910, Novus Biologicals) or Raptor Polyclonal antibody (#20984-1-AP, Proteintech Group) overnight at 4°C. The immunoprecipitated complexes were collected in 50 µl elution buffer containing 0.1M Tris, pH 8 neutralization buffer. For the Raptor antibody immunoprecipitated immunoblot, the acetylated-lysine mouse mAb (Ac-K-103) (#9681, Cell Signaling Technology) was added to the PVDF membrane overnight at 4°C. After washing with PBST, horseradish peroxidase-conjugated secondary antibody (**Table S2**) was added and incubated for 1h. After washing again with PBST, protein bands were visualized using a chemiluminescent substrate for quantitative chemiluminescent western blots (Radiance Plus, #AC2103, Azure) on an Azure Biosystem (Azure c600). RAPTOR or GAPDH antibody was used as an internal control. The protein band was quantified by densitometry using Image J software. The fold change was calculated using the control as background.

### Immunoblotting

For the immunoblot analysis, the proteins were extracted with RIPA buffer (Santa Cruz Biotechnology USA, sc-24948A) containing Protease Inhibitor Tablets (Pierce, #A32963). Protein concentration was determined by BCA Protein Assay (Pierce, #23225). 10μg protein per well was subjected to 4-20% SDS-PAGE. After transfer to a Immobilon®-P PVDF Membrane (#IPVH00010, Millipore-Sigma). The membrane was blocked in 5% BSA/PBST (0.1% Tween-20) for 30 min. The primary antibody (**Table S2**) was added to the PVDF membrane overnight at 4°C. After washing with PBST, horseradish peroxidase-conjugated secondary antibody (**Table S2**) was added and incubated for 1h. After washing again with PBST, protein bands were visualized by chemiluminescent substrate for quantitative chemiluminescent western blots (Radiance Plus, #AC2103, Azure) on an Azure Biosystem (Azure c600). GAPDH antibody was used as an internal control. The protein band was quantified by densitometry using Image J software. The fold change was calculated using the control as background.

### Immunohistochemistry staining

Paraffin section slides of liver tissue were incubated at 65°C for 30 min. After de-paraffinization with xylene 5 min × 2, the slides were rehydrated with a gradient ethanol series from 100%, 95%, 70%, to 50% and then rinsed with deionized H2O. 0.1% Trion X-100 was used for permeabilization, and 5% donkey serum in 3% BSA/PBS was used to block non-specific binding.

The slides were incubated with CD36 (#MA5-32433, Thermofisher) at 4°C overnight, followed by washing with PBS and incubation with substrate (#SK-4285, ImmPACT® AMEC Red Substrate Kit, Vector Labs). The nuclei were counterstained with Hematoxylin Stain Solution, Gill 3 (#MER 347961GL, Mercedes Scientific), and images were captured using a bright-field Olympus microscope.

### Generation of the stably overexpressed CD36 Huh7 cell line

Huh7 cells were maintained in Dulbecco's Modified Eagle Medium (DMEM) (#11995-065, Gibco) supplemented with 10% of Fetal Bovine Serum (FBS) (#12306C, Millipore Sigma) and 1% of Penicillin-Streptomycin (#15140-122, Gibco) at 37°C in a humidified atmosphere with 5% CO_2_ (v/v). The medium was replaced every 3 days if not otherwise stated. For the establishment of CD36 overexpression line, Huh7 cells were transfected with human CD36 cDNA ORF plasmid (C-DYKDDDDK, FLAG; #HG10752-CF, Sino Biological) and vehicle using Lipofectamine 3000 Transfection Reagent (# L3000001, ThermoFisher). The cells were selected with hygromycin (0.5 mg/ml) for two weeks and then harvested for immunoblotting against FLAG to validate overexpression.

### Duolink Proximity Ligation Assay (PLA)

### PLA was performed using Duolink® In Situ Red Starter Kit Mouse/Rabbit (DUO92101, Sigma) according to manufacturer’ instructions. In brief, frozen rat liver tissue sections (5 µm) were soaked in PBS. Subsequently, slides were incubated with the DUOLINK Blocking Stock reagent diluted 1∶5 in Antibody Diluent for 30 min at 37°C. Primary antibodies (anti-TSPO (anti-rabbit, generated in Dr. Papadopoulos’s lab), anti-CPT1A (ab128568), or anti-TSPO (#68137-1-Ig, PTG), anti-ACBP/DBI (#A13293, Abclonal) (**Table S3**) were applied for PLA overnight at 4°C in a humidified chamber. Afterward, slides were washed twice for 5 min each in 1X wash buffer A at room temperature, followed by incubation with PLUS and MINUS PLA probes (1:5 in Duolink® Antibody Diluent) for 30 min at 37°C. Ligation and amplification steps were carried out according to the manufacturer’s instructions. Finally, coverslips were mounted using Duolink In Situ Mounting Media with DAPI, and images were acquired using a ZEISS LSM 880 with an Airyscan confocal microscope equipped with a 63× Oil objective.

### Seahorse assay

A Seahorse assay was conducted utilizing the Agilent Seahorse XF Cell Mito Stress Test Kit (#103015-100, Agilent) and Seahorse XFe96 Analyzer in accordance with the manufacturer’s protocols. In brief, the hepatocytes were isolated from WT or *Tspo* KO rats as described above. The freshly isolated hepatocytes (13000cell/well) were cultured overnight in PDL-coated Seahorse XF Cell Culture Microplates (#103730-100, Agilent). The cells were then treated with either BSA (Veh) or 0.5mM OA for 24hrs. Meanwhile, sensor cartridges were pre-soaked with 200 µl/well of Molecular Biology Grade Water (#46-000-CI, Corning) overnight at 37°C. The next day, the medium for these primary cells was replaced with Agilent Seahorse XF DMEM Medium (#103575-100, Agilent), supplemented with 1 mM pyruvate (#103578-100, Agilent), 2 mM glutamine (#103579-100, Agilent), and 10 mM glucose (#103577-100, Agilent). The cells were then incubated at 37°C in a non-CO2 incubator. For the sensor cartridge, the water was carefully replaced with prewarmed XF Calibrant (#100840-000, Agilent) and incubated at 37°C for 1 h. Subsequently, 20 µl of 100 µM oligomycin, 22 µl of 100 µM Carbonyl cyanide-4 (trifluoromethoxy) phenylhydrazone (FCCP), and 25 µl of 50 µM Rotenone and Antimycin were loaded into port A, B, or C of the sensor cartridge, respectively. An XF Cell Mito Stress Test template was employed to assess the oxygen consumption rate (OCR) of the cells. The OCR data was normalized using the protein amount per well determined by a Bradford assay (#E530, VWR). Results were analyzed utilizing GraphPad Prism 9.5.1 software.

### Mitochondrial Membrane Potential

JC-10 Mitochondrial Membrane Potential Assay Kit (#ab112134, abcam) was used to detect the mitochondrial membrane depolarization of cells. The absorbing value of JC-10 lies on the polarization level of the mitochondrial membrane. Hyperpolarized mitochondria were indicated by increased red fluorescence with higher accumulation of JC-10 compared to depolarized mitochondria stained with green fluorescence. For the primary cells, 13000 cells/well were cultured in PDL-coated 96-well plates. For the cell lines, 10000 cells/well were cultured in 96-well plates. After treatment with either BSA (Veh) or OA (0.5 mM), the cells were washed once with PBS and then incubated with 50 μL/well 1x JC-10 dye-loading solution for 30 min while protected from light. After incubation, 50uL/well of Assay Buffer B was added to each well. The fluorescence intensities at Ex/Em = 490/525 nm (cut off at 515 nm) and 540/590nm (cut off at 570 nm) were monitored. Images were acquired with an inverted fluorescence microscope (REVOLVE, Echo, USA).

### Autophagic flux

An assay using tandem fluorescent-tagged LC3 (ptfLC3 [mRFP-EGFP-LC3], #21074, Addgene) construct was performed to monitor autophagic flux based on the differential pH stability of EGFP and mRFP fluorescent proteins. The cells (primary rat hepatocytes isolated from WT or *Tspo* KO, or Huh7 and CD36_OE Huh7) were treated with either BSA (Veh) or 0.5mM OA for 24hrs, followed by transfection of ptfLC3 [mRFP-EGFP-LC3. After 24 hours, the cells were fixed. The cell nuclei were stained with DAPI. Transfection of the plasmid enables the detection of autophagosomes as yellow puncta (merged signals of mRFP and EGFP labels) and autolysosomes as red puncta (RFP signal alone after quenching EGFP signals by acidic environment [pH 4~5] or acidic lysosomes). Using a ZEISS LSM 880 with Airyscan confocal microscopy, images were captured after 24h treatment of the cells. For each sample, 30 images were taken at a magnification of 630×, and pixel counts quantified areas of yellow or red puncta after subtracting background fluorescence using a cut-off threshold in Image J.

### Acetyl-Co-A (AcCoA) assay

AcCoA was determined using a fluorometry-based assay (#MAK039, Sigma-Aldrich) per manufacturer's instructions. Briefly, 200–500 µg protein of each sample was used. 2 µl of 1M perchloric acid/mg was added to each sample. The samples were homogenized using TissueLyzer II (Qiagen, USA) thoroughly, followed by centrifugation at 10,000×g for 10 min to remove insoluble material. The supernatant was neutralized with 3M potassium bicarbonate solution while vortexing until bubble evolution ceased. After verifying pH was 6–8, the samples were spun to pellet out the potassium bicarbonate. The liver lysates were then incubated with assay mix containing AcCoA substrates, conversion enzyme mix, and fluorescent probe detector. Reactions were performed in triplicate in 96-well plates at 37°C for 15 min, and fluorescence intensities were measured at λ_ex_ = 535/λ_em_ = 587 nm. The readings were normalized to input protein content.

# **Reference**

1. Fischer, A. H., Jacobson, K. A., Rose, J., and Zeller, R. (2008) Hematoxylin and eosin staining of tissue and cell sections. *CSH Protoc* **2008**, pdb prot4986

2. Severgnini, M., Sherman, J., Sehgal, A., Jayaprakash, N. K., Aubin, J., Wang, G., Zhang, L., Peng, C. G., Yucius, K., Butler, J., and Fitzgerald, K. (2012) A rapid two-step method for isolation of functional primary mouse hepatocytes: cell characterization and asialoglycoprotein receptor based assay development. *Cytotechnology* **64**, 187-195
